# Supplementary material for: Mechanisms governing the pioneering and redistribution capabilities of the non-classical pioneer PU.1
Source: Nat Commun. 2020 Jan 21;11:402. doi: 10.1038/s41467-019-13960-2 (PMC6972792; doi:10.1038/s41467-019-13960-2)
Supplement: Supplementary file 7 — Source data [file 41467_2019_13960_MOESM7_ESM.zip › Source_Data/FigureS2/FigureS2B_MotifScanOutput/KmeansCluster8/homerResults/motif39.similar.html]

motif39

## Information for motif39

C
G
A
T
T
A
C
G
C
G
T
A
A
C
T
G
G
T
C
A
C
G
T
A
G
A
T
C
A
C
G
T
T
A
G
C
C
G
T
A
A
T
G
C
G
A
C
T
A
G
T
C
C
G
T
A
  
Reverse Opposite:  

C
G
A
T
T
A
C
G
C
G
T
A
A
T
C
G
A
C
G
T
A
C
T
G
C
G
T
A
C
A
T
G
C
G
A
T
A
C
G
T
G
T
A
C
A
C
G
T
A
T
G
C
G
C
T
A
  

|  |  |
| --- | --- |
| p-value: | 1e-16 |
| log p-value: | -3.813e+01 |
| Information Content per bp: | 1.725 |
| Number of Target Sequences with motif | 35.0 |
| Percentage of Target Sequences with motif | 2.31% |
| Number of Background Sequences with motif | 150.4 |
| Percentage of Background Sequences with motif | 0.33% |
| Average Position of motif in Targets | 116.2 +/- 57.4bp |
| Average Position of motif in Background | 105.3 +/- 73.0bp |
| Strand Bias (log2 ratio + to - strand density) | 0.3 |
| Multiplicity (# of sites on avg that occur together) | 1.00 |
| Motif File: | file (matrix) reverse opposite |

### Similar de novo motifs found

|  |  |  |  |  |  |  |  |
| --- | --- | --- | --- | --- | --- | --- | --- |
| Rank | Match Score | Redundant Motif | P-value | log P-value | % of Targets | % of Background | Motif file |
| 1 | 0.884 | C T A G C G T A A C T G C T G A C G T A A G T C A C G T A T G C C G T A A T G C A C G T A T G C G C T A A G C T | 1e-15 | -36.173062 | 1.79% | 0.19% | motif file (matrix) |
| 2 | 0.916 | A T C G C G T A A C T G T C G A C G T A A T G C A C G T A T G C C G T A A T G C A G C T A T G C G T C A | 1e-14 | -34.331506 | 3.44% | 0.86% | motif file (matrix) |
